# Investigating the Probiotic Potential of Vegan Puree Mixture: Viability during Simulated Digestion and Bioactive Compound Bioaccessibility

**DOI:** 10.3390/nu16040561

**Published:** 2024-02-18

**Authors:** Kübra Küçükgöz, Marcin Kruk, Danuta Kołożyn-Krajewska, Monika Trząskowska

**Affiliations:** Department of Food Gastronomy and Food Hygiene, Institute of Human Nutrition, 3702-776 Warsaw, Poland; marcin_kruk@sggw.edu.pl (M.K.); danuta_kolozyn-krajewska@sggw.edu.pl (D.K.-K.); monika_trzaskowska@sggw.edu.pl (M.T.)

**Keywords:** bioactive compounds, digestion, fermentation, lactobacillus, probiotic, vegan

## Abstract

This study aimed to develop a fermented puree mixture containing plant-based ingredients and potential probiotic strains *Lacticaseibacillus rhamnosus* *K3* and *Lactobacillus johnsonii K4.* The survival of potential probiotic strains, changes in sugar and organic acid concentrations, bioaccessibility of polyphenols, and antioxidant capacity after simulated digestion were examined with sensory quality. The mixture of apple puree, chia seeds, and oat bran or oat flakes was fermented. The sensory quality of the puree mixture was assessed by the quantitative descriptive profile (QDP) method. In vitro digestion was simulated using a static gastrointestinal model. Antioxidant capacity and total polyphenol content were analyzed before and after the digestion phases. All samples changed sensory profiles after fermentation. The overall quality was above six out of ten for every product. Fermentation also changed the organic acid composition, with significant increases in lactic, succinic, and acetic acids. After the digestion process, the survival rate remained above 5.8 log_10_ CFU/g. As a result of fermentation with potential probiotics, the bioaccessibility of the total phenolics and antioxidant activity increased. These results showed that the addition of potential probiotic strains increases nutritional value and could help with healthy nourishment habits. This knowledge can guide the development of consumer-satisfying products in the food industry, expanding the probiotic food market with innovative alternatives.

## 1. Introduction

Food products have been extensively researched to determine whether they enhance consumers’ health and prevent diseases [1]. Probiotics, live microorganisms offering health benefits, are also used for preventing gastrointestinal diseases, antimicrobial activity, and regulating lactose metabolism [2,3]. Scientific evidence supports their safety and efficacy. Probiotic-added products are increasingly available in the market, including drinks and snacks generally containing *Lactobacillaceae*, *Bifidobacterium*, *Saccharomyces*, and *Bacillus* strains [4]. Notably, non-dairy matrices, such as cereals and fruits, have gained prominence due to both health and sustainability concerns and the growing appeal of vegan options in the probiotic-containing foods sector [4,5]. It is of great importance that the type of food matrix carrying probiotic microorganisms may affect the survival of probiotics in the gastrointestinal system, their susceptibility to gastrointestinal conditions (acidity, bile, and various enzymes), as well as their functionality in the body [6,7]. Our selected strains for this research were sourced from a study that studied 38 strains from traditional fermented foods as part of a probiotic selection study. These strains were isolated from cucumber and cabbage pickles in households in the central region of Poland and have shown the capability of survival at low pH levels, good tolerance to bile salts, phenol addition, and moderate hydrophobicity. *L. johnsonii K4* even stands out among these strains due to its ability to multiply under phenol exposure and a hydrophobicity exceeding 60%. This makes *L. johnsonii K4* a promising probiotic strain for further investigation and potential inclusion in functional foods [8].

Fruit, grain, and oil seed mixtures are tasty, nutritious puree mixture choices that are widely consumed around the world. Puree mixtures containing oats, apples, and chia seeds, with the addition of potential probiotic strains, improve the nutritional value of these selected ingredients. The antioxidants in whole oat grains refer to health benefits. Specifically, oat bran includes high amounts of antioxidants in comparison to other parts of grain such as potent avenanthramides [9,10]. In addition to the beta-glucan content, this cereal is a potential source of prebiotics, which stimulate the growth of beneficial microorganisms in the colon [11,12]. Regarding fruits, apples have been studied as a suitable carrier for probiotic microorganisms due to their nutritional properties and food matrix function. Apples, with their prebiotic content, are a good carrier of probiotic strains with amounts of pectin [13]. According to research, functional coatings with probiotic and prebiotic compounds have been applied with success on sliced apples [14,15,16]. Chia seeds have been used as a dietary supplement and in the production of bars, cereals, and cakes [17], while chia seed flour has been used to formulate gluten-free bread with high nutritional value [18]. Fermentation technology is used all around the world for developing functional foods, as it can improve sensory and nutritional qualities and process primary food substrates to remove undesirable compounds [19]. Additionally, fermentation methods have been targeted and developed to enable the synthesis of biologically active metabolites using the right microbial target and suitable substrate for diverse functional purposes. Lactic acid bacteria (LAB)-based fermentation contributes to inadvertently improving the nutritional value and digestibility of various foods, decreasing lactose intolerance, and controlling possible infections [20,21]. Soluble sugars and organic acids are also crucial factors that contribute to determining the chemical stability, pH, nutritional value, acceptability, and storage stability of a product [22]. Lactic acid fermentation is a process that converts and utilizes organic acids found in plant materials, such as LAB, and can decompose the organic acids present in food products and transform them into lactic acid. This process gives a sour taste to the fermented products [23].

Furthermore, fiber and phenolic compounds have been shown to play an important role in colon microbiota interactions [24]. Polyphenols may not always be at their peak biological activity after digestion. Biologically active compounds, which are generally transformed into metabolites during digestion and transported to target organs via blood, have different biological properties from their primary forms [25]. Various methods have been proposed to increase the bioaccessibility of polyphenols and their stability in digestion. One of them is fermentation with LAB, which can convert polyphenols into compounds with higher bioavailability and bioactivity [26]. In addition, LAB can prevent the chemical breakdown of some polyphenols [27]. 

It is essential to examine how digestion affects the survival of potential probiotic strains, bioactive compounds, and antioxidant activity. Moreover, in vitro simulation of gastrointestinal digestion can be used to monitor changes in different parts of the digestive tract [28].

Therefore, this research aims to investigate the survival of potential probiotic bacteria within selected food matrices and assess the bioaccessibility of polyphenols and antioxidant capacity during simulated digestion. Furthermore, the research aims to evaluate the sensory quality of a developed puree mixture incorporating plant-based ingredients and a potential probiotic strain.

## 2. Materials and Methods

### 2.1. Selection of the Probiotic Strains

Potential probiotic strains were obtained from the collection of the “Department of Food Gastronomy and Food Hygiene, Warsaw University of Life Sciences in Poland”. A few strains of *L. brevis*, *L. casei*, *L. rhamnosus*, and *L. johnsonii* were tested, and, due to optimal acidity and acceptable sensory changes on the products after fermentation, *L. rhamnosus* K3 and *L. johnsonii* K4 were used for further research [8,29]. Before application, bacteria were activated from a frozen culture stored at −80 °C. They were incubated at 30 °C for 24 h in 10 mL of MRS broth (Merck-110.660, Darmstadt, Germany). After completing the incubation period, the tubes were centrifuged at 10,000 rpm for 5 min (laboratory centrifuge MPW-251; MPW MED Instruments, Warsaw, Poland) to separate bacterial cells from the medium. The supernatant was replaced with 8.5 g/kg of saline, and the centrifugation procedure was performed three times to remove residual growth medium.

### 2.2. Development of Potential Probiotic Puree Mixture

The food matrix for fermentation was selected from non-dairy raw materials with high nutritional properties. Apples (“Golden Delicious”), chia seeds, oat bran, and oat flakes were purchased from local markets in Poland. Fresh apples were peeled, washed, and cut into small pieces. The apple pieces were then cooked for five minutes in a pressure cooker with the addition of 200 mL of water for every 1000 g of peeled apples. After cooking, the softened apple pieces were mashed using a high-powered mixer (Bosch ErgoMixx 1000W) to achieve a homogeneous mixture. The ingredients were then mixed in the ratios specified in Table 1. The mixture was pasteurized at 72 °C for 15 min, followed by cooling to room temperature for inoculation. For each sample, a 1 mL (9 log_10_ CFU/g) bacterial solution of *L. rhamnosus* K3 or *L. johnsonii* K4 in saline solution was added to 100 g of the puree mixture and inoculated for 15 h at 30 °C. After inoculation, the samples were immediately cooled to 4 °C and stored in the refrigerator for 24 h before further analysis.

### 2.3. In Vitro Gastrointestinal Digestion (GIS)

In vitro digestion studies play a pivotal role in evaluating the bioaccessibility of nutrients and bioactive compounds within diverse food matrices. These studies emulate the human digestive process, simulating oral, gastric, and small intestinal phases to understand changes in food components during digestion. The methodology was completed according to Minekus et al. (2014) [30]. Oral phase was started by combining 3.5 mL simulated salivary fluid (pH 7) with 5 g of the food sample and then adding 0.5 mL amylase solution (75 U/mL) from *Aspergillus oryzae* (Sigma-Aldrich, Lot SLCD1111, Poznan, Poland), 25 g CaCl_2_ (0.3 M) (Chempur, Piekary Śląskie, Poland), and 975 mL water.

The resulting mixture, to represent the oral phase, underwent grinding with 2.5 mL CaCl_2_ (0.3 M (Chempur, Piekary Śląskie, Poland)) and 975 mL distilled water. Lastly, pH was adjusted to 7 with NaOH (1 M) (Chempur, Piekary Śląskie, Poland) and shaken for 2 min at 37 °C and 100 rpm. Gastric phase was started by adjusting the mixture’s pH to 2.5, then 7.5 mL simulated gastric juice was added, including 1.6 mL pepsin from porcine gastric mucosa (2000 U/mL) (Sigma Aldrich, Lot SLBH3879V, Poznan, Poland), 5 μL CaCl_2_ (0.3 M) (Chempur, Piekary Śląskie, Poland), and 690 μL distilled water. The mixture was shaken for 2 h at 37 ± 2 °C and 100 rpm. Small intestinal phase began with combining the gastric chyme with 5.5 mL simulated intestinal fluid (pH 7) and then 2.5 mL pancreatin from porcine pancreas (800 U/mL) (Sigma Aldrich, Lot SLBX1822, Poznan, Poland), 1.25 mL porcine bile extract (10 mM) (Sigma Aldrich, Lot SLCJ7934), 20 μL CaCl_2_ (0.3 M), and 585 μL distilled water were added. Finally, pH was maintained at 7 with NaOH (1 M) and incubated for 2 h at 37 ± 2 °C and 100 rpm.

### 2.4. Microbiological Viability Analysis

For the analysis of *L. rhamnosus K3* and *L. johnsonii K4* CFUs, 1 g of the puree mixtures with probiotics was added to 9 mL of sterile peptone water and homogenized with a stomacher at medium speed for 2 min. Seven-fold serial dilutions were prepared from the homogenized samples with peptone (Sigma-Aldrich, Poznań, Poland) water. Appropriate dilutions were inoculated onto MRS Agar (pH 6.8 ± 7.2, Merck-110,660, Darmstadt, Germany), and Petri plates were incubated at 37 °C for 48 h. After the digestion in the gastric and intestinal phases, an aliquot of the digested sample was decimally diluted with peptone water and plated onto MRS agar plates at 37 °C for 48 h. Colony counts were calculated as CFU/g of mixtures, and the obtained means of the data were transformed to log_10_ CFU/g.

### 2.5. Bioactive Properties’ Determination

#### 2.5.1. 12,2′-Azino-Bis(3-ethylbenzothiazoline-6-sulfate) (ABTS•+) Radical Cation Depolarization Assay

ABTS stock solution was dissolved in sodium acetate–acetic acid buffer (20 mM, pH 4.5) to make a 7 mM ABTS stock solution. A total of 20 mL of distilled water was added to 0.0256 g of potassium persulfate (K_2_S_2_O_8_) (weighed to an accuracy of 0.0001 g). To 0.0384 g of ABTS radical reagent (2′2-zinobis-3-ethylbenzothiazoline-6-sulfonic acid) (weighed to the nearest 0.0001 g), 5 mL of distilled water was added, followed by 5 mL of the previously prepared aqueous potassium persulfate solution. The solution was prepared at least 12 h before the planned determination. The solution was stored at room temperature and protected from light. Before the assay, the absorption of radical solution in PBS was adjusted to absorbance 0.7. For carrying out the assay, 50 μL of the prepared sample dilution was added to a polystyrene plate (96 well, 300 μL), and 150 μL of ABTS radical solution was added in PBS. Measurements were performed exactly after the sample had been incubated with ABTS for 6 min at room temperature. The absorbance at a wavelength ÿ = 734 nm was measured using a microplate reader. The result is expressed as mg of ascorbic acid per a given volume or mass of the material tested. The calculation of the result was based on the 10-point standard curve created by ascorbic acid (Poch, Gliwice, Poland).

#### 2.5.2. Total Polyphenolic Content Determination

For the determination of total polyphenolic content, the colorimetric method with the use of the Folin-Ciocalteu reagent was used. The reaction was carried out in an alkaline environment by using an anhydrous sodium carbonate solution in which the phenolate anion reduces the molybdenum. The intensity of the color produced is proportional to the total amount of phenolic compounds. Total polyphenols are expressed as gallic acid equivalent (mg GAE) based on the weight or volume of the sample. For carrying out the assay, 20 μL of the previously prepared sample dilution was added to a polystyrene plate (96 wells, 300 μL), 100 μL of Folin-Ciocalteu reagent was added, and it was left for 5 min at room temperature in a dark place. An amount of 80 μL of sodium carbonate solution was poured into the wells, mixed at 150 rpm for 5 min, and left for 2 h in the dark. The samples were mixed for one minute at 150 rpm on a reading machine before measurement. The absorbance was measured at a wavelength ÿ = 750 nm using a microplate reader. The calculation of the result was based on the 10-point standard curve created from gallic acid (Merck, Poznań, Poland).

### 2.6. Organic Acids and Sugar Detection

Before the analysis, samples were diluted in deionized water at a 1/10 ratio and then centrifuged for 15 min at 10,000 rpm using an Eppendorf Centrifuge 5804 R (Hamburg, Germany). Then, 1 mL of the samples was firstly filtered with 0.45 μm syringe PES filter into the vials. Organic acids and sugars were analyzed with an HPLC system (Shimadzu, USA Manufacturing Inc, USA, consisting of two LC-20AD pumps, a CBM-20A controller, a CTD-20AC oven, a SIL-20AC autosampler, RID-10A detector, and UV/Vis SPD-20AV detector). For the separation of related compounds, we used Aminex HPX-87H column 300 × 7.8 mm (Bio-Rad, USA) at 40 °C with a flow rate of 0.5 mL/min and a mobile phase of 10 mM H_2_SO_4_. Quantification was based on the detection of each analyte at 210 nm wavelength using UV/Vis, RI, and external standard curves ranging from 0.12 to 40 μg per injection.

### 2.7. Bioaccessibility Index

In this study, the bioaccessibility index of phenolic compounds was calculated according to [31,32], based on the equation below:

BI (%) = A/B × 100

where A is the quantification of total phenolic content or antioxidant capacity (ABTS) after in vitro digestion; B is the quantification of total phenolic content or antioxidant capacity (ABTS) puree mixture.

### 2.8. Sensory Evaluation

The quantitative descriptive profile (QDP) method was utilized to objectively determine the sensory quality of the produced puree mixture. This procedure is in accordance with ISO Standard 13299:2016 [33], which provides general guidance for establishing a sensory profile. A linear graphical scale (100 mm) was used and converted to numerical values (0 to 10 conventional units). A list of descriptors was chosen and defined during the panel discussion and then verified in the preliminary session. Most of the tested attributes were measured on a scale from no intensity to high intensity, with an overall quality rating ranging from very low to very high. The trained panel consisted of 10 assessors, each with 4 to 12 years of experience in sensory evaluation, a good understanding of sensory methodology, and familiarity with the sensory quality being evaluated. To achieve this objective assessment, a set of 11 sensory descriptors was carefully selected and defined, as outlined in the supplementary materials (Table S1). To prepare the samples for evaluation, 50 mL transparent containers with lids were used, and each sample was assigned a unique 3-digit code and served randomly to the experts at room temperature. To ensure neutrality between samples, still water was provided as a neutralizer after each sample. Overall, these measures ensured a scientifically rigorous and standardized approach to the evaluation of the sensory quality of the produced puree mixture. An overall quality rating was determined on a scale of 0 to 10, where a rating below 6 was considered “poor”, 6 to 7 was considered “fair”, and 8 to 10 was considered “good” [34].

### 2.9. Statistical Analysis

The statistical analyses were conducted using Statistica 13.3 (StatSoft, Kraków, Poland). Standard deviation (SD) and arithmetic mean were calculated. The data were analyzed by multivariate analysis of variance (ANOVA) and Tukey HSD post hoc test. In order to analyze the sensory analysis results, principal component analysis (PCA) was conducted using a correlation matrix. The difference was considered statistically significant when *p* < 0.05 in relation to the count of bacteria, the results of chemical analyses, pH, and the results of sensory evaluation. Error bars in numbers and values after “±” in tables represent SD. All laboratory analyses were performed in triplicate.

## 3. Results and Discussion

### 3.1. Evaluation of the Survival of L. rhamnosus K3 and L. johnsonii K4 in Puree Mixtures during Simulated Digestion

It is recommended that for food to have a therapeutic effect, between 10^6^ and 10^8^ CFU/g or mL of viable probiotic cells should remain after the intestinal digestion stage [35]. The survival of probiotic bacteria during oral and gastric digestion is a crucial factor in their effectiveness in delivering health benefits. In order to assess the survival of potential probiotics added to puree mixtures, a study was conducted to evaluate the survival of *L. rhamnosus K3* and *L. johnsonii K4* in puree mixtures during simulated gastric and intestinal digestion. The initial number of probiotic bacteria in the puree mixtures was found to be 9 log_10_ CFU/g for both *L. rhamnosus K3* and *L. johnsonii K4.* After undergoing simulated gastric digestion, a significant decrease in the total counts of both strains was observed. However, during the simulated intestinal phase, there was no significant change in the counts of *L. rhamnosus K3* and *L. johnsonii K4* compared to the gastric phase, except for sample BR3, which showed growth during the intestinal phase, although this growth was not statistically significant. The numbers of *L. rhamnosus K3* in the puree mixtures samples were detected as ranging from 9.1 to 9.2 log_10_ CFU/g, while the numbers of *L. johnsonii K4* in the puree mixtures samples were detected as ranging from 9.13 to 9.20 log_10_ CFU/g. These findings suggest that the probiotics added to the puree mixtures were able to survive the simulated gastric and intestinal digestion and maintained their viability during the process (Table 2).

It was found that the *L. rhamnosus K3* strain with samples of oat bran survived at 6.05 log CFU/g under gastric digestion and 5.94 log CFU/g with samples of flakes, with an increase to 6.20 log_10_ CFU/g for the bran samples and a slight decrease for the flake samples to 5.80 log_10_ CFU/g during the intestinal phase. Similarly, for *L. johnsonii K4*, a higher total count of 6.30 log_10_ CFU/g was observed after gastric digestion for samples with bran, and a higher recovery after the intestinal phase was also observed with bran samples after the intestinal phase (6.03 log_10_ CFU/g). Overall, these findings showed that both *L. rhamnosus K3* and *L. johnsonii K4* had better survival rates during the digestion process when combined with oat bran compared to flakes. This result can be related to oat bran’s high protein and fiber content. In another research study on the different oat compounds’ effects on the gut microbiome, oat bran showed the most positive effect on the growth of Bifidobacterium [36].

The results of the study help us to understand how important it is for microbes to survive during digestion. One study conducted by Emser et al. (2017) [15] investigated the survival of *Lactobacillus plantarum* incorporated in apple cubes during simulated in vitro digestion. The results showed that *L. plantarum* survived the quick simulation of gastrointestinal digestion for 2 h and was not reduced. The study suggested that apple-based products can provide a protective matrix for probiotic bacteria during digestion, which can improve their survival. Other research examined the viability and survival of *L. paracasei* in dehydrated apple slices as a carrier. The researchers observed the successful adherence of the bacterial strain to the apple matrix and its ability to tolerate the harsh conditions of the gastrointestinal (GI) tract. The bacterial load in the dehydrated apple slices met the recommended threshold of more than 7 log_10_ CFU/g for a probiotic product. After simulated digestion, the survival rate of *L. paracasei* remained high, with only a 2 log_10_ CFU/g reduction in bacterial cells. These findings suggest that dehydrated apple slices can effectively deliver viable probiotic cells to the gut [37]. Another study focused on *L. salivarius* spp. *salivarius* as the bacterial strain and investigated its survival rate after digestion using encapsulation. The study compared the viability of encapsulated and non-encapsulated forms of *L. salivarius* spp. *salivarius* in dried apple during simulated gastrointestinal digestion. The researchers found that the encapsulated form exhibited higher resistance to the GI simulation than the non-encapsulated form. Encapsulation improved the survival rate and total microorganism content of *L. salivarius* spp. *salivarius* compared to the non-encapsulated form. However, both encapsulated and non-encapsulated forms showed a decrease in survival with storage time during the GI stages, highlighting the importance of freshness and timely consumption for optimal probiotic effects [38].

All these studies contribute to our understanding of how apple-based matrices can serve as carriers for probiotics and enhance their survival during digestion. Among factors that are relevant to their studies are the strain of probiotics, the form of delivery (e.g., puree mixtures, dehydrated apple slices, encapsulation), and the simulated digestion conditions. The acidic pH of apples offers a potentially favorable environment for probiotic strain growth. Moreover, apples have a rich source of prebiotics, including soluble fibers like pectin, which can act as substrates for beneficial bacteria within the gastrointestinal tract. Prebiotic fibers in apples promote a balanced gut microbiota by improving probiotic populations [37]. Based on the findings, oat bran in particular can protect potential probiotic bacteria in apple-based matrices, allowing them to survive gastrointestinal conditions.

### 3.2. Evaluation of the Bioactive Components of Fermented and Non-Fermented Puree Mixtures during Simulated Digestion

The bioaccessibility of dietary polyphenols, despite their presence in plant-based foods, is often limited by several factors, including their large molecular size, molecular weight, polarity, shape, and susceptibility to degradation within the small intestine [39,40]. Furthermore, certain microorganisms possess the capacity to metabolize complex phenolic compounds into bioactive derivatives, thereby enhancing their bioavailability [41]. Apple products that contain probiotic bacteria also contain polyphenols, which may improve gut health and immunity. It is crucial to understand whether these polyphenols and antioxidants are bioaccessible within the gastrointestinal tract to show their health benefits. The amount of TPC in puree mixtures with oat flakes and oat bran decreased during the digestion process. Before digestion, in the initial phase, non-fermented samples with oat bran and fermented samples with oat bran were significantly higher than the other samples. Notably, for the fermented mixtures, TPC decreased less. The fermentation of the puree mixtures probably favored the positive results achieved in this study. The TPC was significantly increased for BR3, BRC, and FLC samples, and for the FL4 and FL3 samples, TPC amounts did not change significantly in the last stage of digestion, the intestinal phase. In this stage, non-fermented samples showed a significantly lower amount of TPC compared with fermented samples (*p* < 0.05). It is particularly interesting to use LAB fermentation as a way of increasing the bioaccessibility of polyphenols. The promising results for improved bioactivity of phenolic extracts pretreated with LAB have been demonstrated (Table 3).

Fermented samples have a higher bioaccessibility of total polyphenol content at the end of the intestinal digestion; for example, non-fermented samples with oat bran’s bioaccessibility was 29.30%, while a sample fermented with *L. rhamnosus K3* was 44.5% and with *L. johnsonii K4* was 46%. The same scenario also applied to samples with oat flakes: non-fermented samples’ bioaccessibility was 34%, while samples fermented with *L. rhamnosus K3* were 40.6% and with *L. johnsonii K4* were 50.3% (Table 3). Additionally, the stability of phenolic compounds may be linked to the production of lactic acid during fermentation [41]. For instance, the fermentation of various fruit and vegetable juices using specific LAB strains, including *L. plantarum* ASCC 292, *L. brevis* 145, *Weissella cibaria* 64, *Leuconostoc mesenteroides* 12b, *L. brevis* POM4, and *Weissella confusa* LK4, has been reported to enhance their antioxidant activities [42].

### 3.3. Evaluation of the Organic Acids of Fermented and Non-Fermented Puree Mixtures during Simulated Digestion

**Figure 1:**
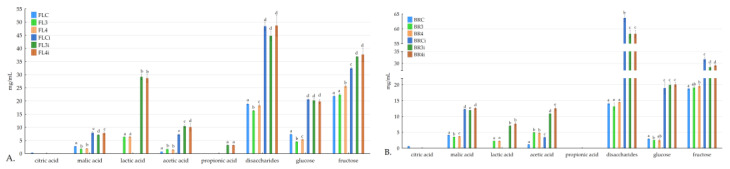
Organic acids and sugars before and after digestion in the analyzed samples; BRC: control sample with oat bran, FLC: control sample with oat flakes, BR3 and FL3: puree mixtures with *L. rhamnosus K3* fermentation, BR4 and FL4: puree mixtures with *L. johnsonii K4* fermentation, FLCi, FL3i, FL4I, BRCi, BR3i, and BR4i: samples after digestion; (**A**) samples with oat flakes; (**B**) samples with bran; a, b, c, d, and e mean the statistical difference between the samples in the post hoc Tukey’s test (*p* < 0.05); error bars mean standard deviation; *n* = 3.

The levels of sugars and organic acids present in fermented food are important indicators of the activity of microorganisms during fermentation. Specific organic acids, including malic, succinic, lactic, and acetic, are found after fermentation and in in vitro digestion (Figure 1). Before fermentation, the control samples (BRC, FLC) exhibited the presence of citric acid, malic acid, and acetic acid. After lactic acid fermentation, significant changes in the organic acid composition were observed. All fermented samples (BR3, BR4, FL3, and FL4) showed the presence of malic acid, lactic acid, acetic acid, and, in some instances, propionic acid. Malic acid and acetic acid were significantly higher in fermented samples with oat bran (BR3 and BR4) than in fermented samples with oat flakes (FL4 and FL3). Lactic acid, which is produced by lactic acid bacteria during carbohydrate metabolism, increases as expected during fermentation. Similar observations have been made in other lactic acid bacteria-fermented products, highlighting the importance of residual sugars in maintaining probiotic activity in fermented foods [42]. In another research study on apple juice after lactic acid fermentation, the organic acid profile changed to become mostly malic acid [43]. The concentration of acetic acid also increased significantly during fermentation. This could be explained again with LAB energy metabolism turning sugars into lactic acid and also other metabolites such as acetic acid [44]. The citric acid concentration was only detected in non-fermented samples and could not be detected after fermentation. It was also similar for fermented pomegranate juice with *L. acidophilus* and *L. plantarum*, which led to a significant decrease in citric acid levels. This phenomenon can be explained as LAB can utilize citric acid for carbon sourcing [45]. Upon in vitro digestion, further alterations in organic acid composition were observed. All detected organic acid content increased significantly after digestion, suggesting its stability under gastrointestinal conditions. Additionally, propionic acids were detected in the digested samples (FL3i and FL4i), indicating the generation or liberation of these acids during digestion. A similar behavior was also observed in the studies working on different vegetables and fruits [46,47].

After in vitro digestion, there was a significant increase in the concentrations of disaccharides, glucose, and fructose in all samples. The increase in sugars after digestion can be attributed to the breakdown of complex carbohydrates, such as starch and polysaccharides, present in the food matrix. When these complex carbohydrates are subjected to the digestive process, they undergo enzymatic hydrolysis, leading to the release of simpler sugars such as glucose and fructose [48].

### 3.4. Sensory Evaluation

**Figure 2:**
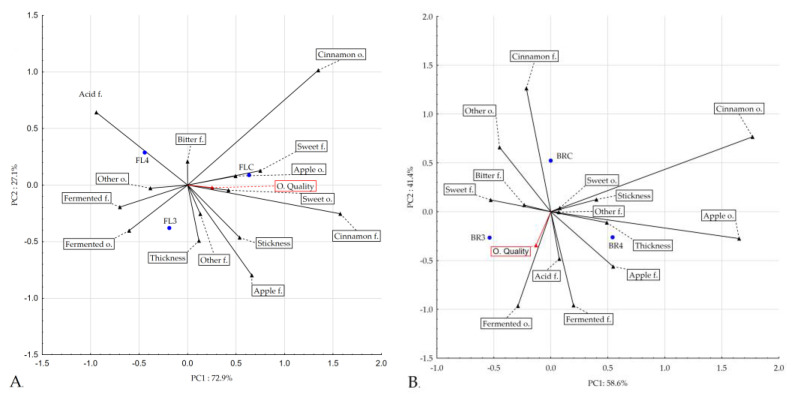
PCA of the puree mixture samples with oat flakes (**A**) and the PCA of the puree mixture samples with oat brans (**B**).

In this study, a potential probiotic-enriched puree mixture was prepared as an alternative functional food for human consumption. It is therefore crucial that fermented probiotic puree mixtures have acceptable sensory properties. Table 4 shows the sensory evaluation results of the puree mixtures enriched with potential probiotics. The fortification of oat flakes and oat bran, including samples with *L. rhamnosus K3* and *L. johnsonii K4*, provided acceptable sensory properties of puree mixtures after probiotic addition. The research also focuses on the addition of a potential probiotic strain called *L. paracasei* ssp. *paracasei* to apple juice formulations and its impact on sensory characteristics. In this study, the probiotic strain was added to apple juice in order to determine whether it affected appearance, aroma, texture, or purchase intent. When evaluated for these sensory attributes, there was no significant difference (*p* > 0.05) in acceptance between the various formulations, indicating that the inclusion of the probiotic culture did not affect acceptance [49]. Fermentation positively influenced the sensory characteristics of the potential probiotic-added puree mixtures, enhancing acidity and fermented odors and flavors. Samples with oat flakes fermented with *L. johnsonii K4* (FL4) showed the most intense apple, cinnamon, and fermented attributes, making them appealing options for human consumption. Overall, fermentation was a beneficial process in improving the sensory properties of the puree mixtures. The results of the descriptive sensory profile analysis of our products were consistent with this finding. PCA showed common characteristics between the flakes’ control product and fermented samples. A direct effect of fermentation on the enhancement of the acid and fermented odor and flavor characteristics is shown in Figure 2A. As a result of fermentation, the samples changed their sensory profile. The greatest change after fermentation occurred in the range of attributes of cinnamon flavor, which corresponded to the high intensity of cinnamon flavor in the control sample and the low intensity in the fermented samples. This is also confirmed by the correlation obtained between the analyzed determinants in terms of PCA. In the PCA of the flakes, the distinguishing feature of the cinnamon flavor is opposite to the distinguishing features of the odor, the fractionated flavor, and the acid flavor. The results presented in Figure 1B illustrate the common characteristics between the bran control product and fermented samples. The common features were sweetness, apple odor and flavor, and structure characteristics. The fermentation directly affected the acid and fermented flavor characteristics. As a result of fermentation, the samples changed their sensory profile. Also, in this case, as in the bran samples, the greatest change after fermentation occurred in the range of notes of the cinnamon flavor, which corresponded to the high intensity of the cinnamon flavor in the control sample and the lower intensity in the fermented samples. This is also confirmed by the correlation obtained between the analyzed determinants in terms of PCA. In the PCA of the flakes, the distinguishing feature of the cinnamon flavor is opposite to the distinguishing feature of the acid flavor.

## 4. Conclusions

The presented study successfully developed a puree mixture with probiotics added and a suitable food matrix that promotes the growth of *L. rhamnosus K3* and *L. johnsonii K4.* Sensory properties were not compromised, with all samples scoring above six out of ten for overall quality. This suggests the mixture’s potential commercialization in the probiotic food market. During simulated digestion, oat bran samples exhibited higher survival rates under gastric digestion compared to flakes. BR3 and BR4 also showed increased survival during the intestinal phase, suggesting oat bran’s better carrier properties in terms of probiotic survival during digestion. The total polyphenol content in fermented samples was more bioaccessible at the end of intestinal digestion than in non-fermented samples. BR3 and BR4 also had significantly increased bioaccessibility compared to non-fermented bran samples. Similar trends were observed for oat flakes, with fermented samples exhibiting higher bioaccessibility percentages than non-fermented samples. Lactic acid fermentation also changed the organic acid composition of the food matrix. The study observed significant changes in malic, succinic, lactic, and acetic acids, and it may also bring potential health benefits. In conclusion, this study provides insights for developing probiotic-enriched puree mixtures with a suitable food matrix. Probiotic viability, sensory properties, and improved bioaccessibility of phenolic compounds were demonstrated. This knowledge can guide the development of consumer-satisfying products in the food industry, expanding the probiotic food market with innovative alternatives.

## Figures and Tables

**Table 1:** Proportion of the ingredients in the developed puree mixture.

Sample	Ingredients					
	Apple	Oat Flakes	Oat Bran	Chia Seeds	Cinnamon	Probiotic
BRC	100 g	-	10 g	2 g	0.3 g	-
FLC	100 g	10 g	-	2 g	0.3 g	-
BR3	100 g	-	10 g	2 g	0.3 g	*L. rhamnosus K3*
BR4	100 g	-	10 g	2 g	0.3 g	*L. johnsonii K4*
FL3	100 g	10 g	-	2 g	0.3 g	*L. rhamnosus K3*
FL4	100 g	10 g	-	2 g	0.3 g	*L. johnsonii K4*

Explanatory notes: BRC: control sample with oat bran, FLC: control sample with oat flakes, BR3 and FL3: puree mixtures with *L. rhamnosus K3* fermentation, BR4 and FL4: puree mixtures with *L. johnsonii K4* fermentation.

**Table 2:** The count of potential probiotic bacteria in the puree mixtures after fermentation and during the digestion process (log_10_ CFU/g).

Samples	After Fermentation	After Gastric Phase	After Intestinal Phase
BR3	9.10 ^aA^	6.05 ^aB^	6.20 ^aB^
BR4	9.10 ^aA^	6.30 ^aB^	6.03 ^aB^
FL3	9.20 ^aA^	5.94 ^bB^	5.80 ^bB^
FL4	9.10 ^aA^	5.38 ^cB^	5.70 ^bC^

Explanatory notes: Significant differences between samples are represented by means in the same column followed by different lowercase letters, and significant differences between samples are represented by means in the same row followed by different uppercase letters. Tukey HSD test shows that statistical differences in lowercase are applicable to all samples in the same column (*p* < 0.05).

**Table 3:** Data of antioxidant capacity, total phenolic content, and bioaccessibility of puree mixture samples in initial phase (non-digested) and after digestion process.

Samples	Initial Samples TPC (GAE mg/100 g)	Digested Samples TPC (GAE mg/100 g)	Bioaccessibility of TPC	Initial Samples Phase ABTS (VCEAC mg/100 g)	Digested Samples ABTS (VCEAC mg/100 g)	Bioaccessibility of ABTS
BRC	40.9 ± 2.05 ^eA^	12 ± 2.9 ^aC^	29.30%	122.0 ± 5.83 ^cA^	34.7 ± 2.71 ^bC^	28.40%
FLC	33.6 ± 2.74 ^bcA^	11.42 ± 8.9 ^abC^	34.00%	110.2 ± 5.8 ^aA^	36.2 ± 2.88 ^bC^	32.70%
BR3	39.8 ± 4.78 ^deA^	17.7 ± 1.2 ^cC^	44.50%	102.3 ± 5.47 ^aA^	44.48 ± 4.9 ^aC^	43.40%
BR4	36.1 ± 2.01 ^cdA^	16.6 ± 0.1 ^bcC^	46.00%	106.0 ± 3.52 ^aA^	37.3 ± 1.3 ^bC^	35.18%
FL3	31.0 ± 3.27 ^abA^	12.6 ± 1.9 ^cB^	40.60%	78.4 ± 5.01 ^bA^	38.05 ± 2.17 ^bC^	48.50%
FL4	28.8 ± 1.48 ^aA^	14.5 ± 9.8 ^cB^	50.30%	84.1 ± 6.77 ^bA^	36.5 ± 1.03 ^bC^	43.40%

Explanatory notes: BRC: control sample with oat bran, FLC: control sample with oat flakes, BR3 and FL3: puree mixtures with *L. rhamnosus K3* fermentation, BR4 and FL4: puree mixtures with *L. johnsonii* fermentation. Significant differences between samples separately for total polyphenol content and antioxidant activity are represented by means in the same row followed by different lowercase letters, and significant differences between samples are represented by means in the same column followed by different uppercase letters. Tukey HSD test shows that statistical differences in lowercase are applicable to all samples in the same row (*p* < 0.05).

**Table 4:** Intensity of defined attributes for developed puree mixture (0–10 c.u.) (*n* = 26).

Attribute	BRC	FLC	BR3	BR4	FL3	FL4
Apple o.	3.57 ^bc^	4.73 ^a^	5.85 ^c^	5.92 ^a^	6.75 ^ab^	6.87 ^ab^
Cinnamon o.	2.60 ^ac^	3.19 ^a^	4.56 ^b^	5.69 ^a^	6.14 ^b^	6.54 ^c^
Fermented o.	2.01 ^b^	2.12 ^b^	2.93 ^a^	3.44 ^a^	3.49 ^a^	4.08 ^ab^
Sweet o.	2.89	3.34	2.75	2.90	2.79	2.51
Other o.	0.47 ^a^	1.21 ^ab^	1.36 ^ab^	1.81 ^b^	1.93 ^a^	2.06 ^a^
Thickness	5.75	5.88	5.45	6.43	6.38	5.37
Stickiness	4.93 ^ab^	5.24 ^b^	5.85 ^ac^	6.04 ^ab^	6.08 ^ab^	6.23 ^c^
Apple f.	4.67 ^ac^	5.33 ^ab^	5.76 ^abc^	6.40 ^b^	6.52 ^ab^	6.84 ^c^
Cinnamon f.	2.83 ^c^	3.24 ^d^	3.85 ^a^	5.06 ^a^	5.23 ^bc^	7.01 ^ab^
Fermented f.	1.88 ^a^	2.53 ^ab^	3.35 ^a^	3.74 ^a^	3.75 ^a^	3.81 ^a^
Sweet f.	2.22 ^a^	2.96 ^b^	3.28 ^a^	3.43 ^a^	3.52 ^ab^	4.79 ^ab^
Acid f.	1.43 ^a^	1.85 ^a^	2.19 ^a^	2.33 ^a^	2.35 ^a^	4.03 ^b^
Bitter f.	1.21	1.28	1.33	0.86	1.00	1.41
Other f.	1.00	1.06	0.94	2.55	1.22	0.66
Overall Quality	6.16	6.96	6.89	6.63	6.63	6.47

Explanatory notes: c.u.: conventional units, BC: control sample with oat bran, FC: control sample with oat flakes, BR3 and FL3: puree mixtures with *Lacticaseibacillus rhamnosus K3* fermentation, BR4 and FL4: puree mixtures with *L. johnsonii K4* fermentation, o.: odor, f.: favor. Significant differences between samples are represented by means in the same row followed by different lowercase letters (*p* < 0.05).

## Data Availability

Data are contained within the article.

## Supplementary Materials

The following are available online at https://www.mdpi.com/article/10.3390/nu16040561/s1, Table S1: Sensory descriptors and their definitions used in the sensory analysis of breakfast mixture; Table S2: BRC—control sample with oat bran, FLC control sample with oat flakes, BR3 and FL3 puree mixtures with L. rhamnosus K3 fermentation, BR4 and FL4 puree mixtures with L. johnsonii K4, fermentation, FLCi,FL3i,FL4I,BRCi,BR3i,BR4i are samples after digestion. Organic acids and sugars before and after digestion in the analyzed samples; a. samples with oat flakes; b. samples with bran.
