# Supplementary material for: Investigating the Probiotic Potential of Vegan Puree Mixture: Viability during Simulated Digestion and Bioactive Compound Bioaccessibility
Source: Nutrients. 2024 Feb 18;16(4):561. doi: 10.3390/nu16040561 (PMC10893087; doi:10.3390/nu16040561)
Supplement: Supplementary file 1 [file nutrients-16-00561-s001.zip › nutrients-2856695-supplementary.pdf]

Table S1: Sensory descriptors and their definitions used in the sensory analysis of breakfast mixture.

Descriptors	Definition	Anchors
	**Odour descriptors**	
**Apple**	Odour characteristic of apple and apple-based products	"none" to "very intense."
**Cinnamon**	Odour characteristic of cinnamon	"none" to "very intense."
**Fermented**	Odour characteristic of fermented apple	"none" to "very intense."
**Sweet**	The basic differentiator does not require characterization	"none" to "very intense."
**Other**	Odour other than those described above	"none" to "very intense."
	**Texture attributes**	
**Thickness**	A bulky characteristic of a product as sensed in mouth or by feeling and seeing e.g. During pouring of the product.	"very liquid" to "very thick."
**Stickiness**	Adhesion to processing equipment, cohesion of powders, sticking to packaging, and sticking to fingers and parts of the mouth	"imperceptible" to "very stick."
	**Flavour descriptors**	
**Apple**	Flavour characteristic of apple and apple-based products	"none" to "very intense."
**Cinnamon**	Flavour characteristic of cinnamon	"none" to "very intense."
**Fermented**	Flavour characteristic of products subjected to fermentation.	"none" to "very intense."
**Sweet**	The basic differentiator does not require characteristics (flavour characteristic of white sugar)	"none" to "very intense."
**Acid**	Flavour characteristic of products subjected to acids.	"none" to "very intense."
**Bitter**	The basic distinguishing feature does not require characteristics (bitter flavour characteristic of grapefruit)	"none" to "very intense."
**Other**	Enter the intensity and name or associate	"none" to "very intense."
**Overall quality**	Impression based on all testes	"very low" to "very high."

Table S2. BRC—control sample with oat bran, FLC control sample with oat flakes, BR3 and FL3 puree mixtures with *L. rhamnosus K3* fermentation, BR4 and FL4 puree mixtures with *L. johnsonii K4, fermentation,* FLCi,FL3i,FL4I,BRCi,BR3i,BR4i are samples after digestion. Organic acids and sugars before and after digestion in the analyzed samples; a. samples with oat flakes; b. samples with bran.

Sample	Citric Acid (mg/mL)	Malic Acid (mg/mL)	Lactic Acid (mg/mL)	Acetic Acid (mg/mL)	Propionic Acid (mg/mL)	Disaccharides (mg/mL)	Glucose (mg/mL)	Fructose (mg/mL)
**BR3**	0.0 ± 0.0	3.53 ± 0.07	2.3 ± 0.0	4.933 ± 0.057	0.0 ± 0.0	13.167 ± 0.577	2.6 ± 0.0	19.07 ± 0.192
**BR3i**	0.0 ± 0.0	11.9 ± 0.0	7.033 ± 0.057	11.067 ± 0.057	0.0 ± 0.0	58.0 ± 0.67	19.9 ± 0.47	28.43 ± 0.53
**BR4**	0.0 ± 0.0	3.733 ± 0.057	2.3 ± 0.0	4.8 ± 0.057	0.0 ± 0.0	14.467 ± 0.167	2.533 ± 0.167	19.567 ± 0.167
**BR4i**	0 ± 0	12.57 ± 0.19	7.67 ± 0.25	12.87 ± 0.34	11.9 ± 0.25	57.83 ± 0.37	20.13 ± 0.37	28.83 ± 0.37
**BRC**	0.6 ± 0.0	4.133 ± 0.057	0.0 ± 0.0	1.166 ± 0.057	0.0 ± 0.0	14.033 ± 0.167	2.933 ± 0.057	18.733 ± 0.167
**BRCi**	0.0 ± 0.0	12.3 ± 0.0	0.0 ± 0.0	3.433 ± 0.057	0.0 ± 0.0	63.633 ± 0.577	19.6 ± 0.47	31.7 ± 0.472
**FL3**	0.0 ± 0.0	1.767 ± 0.057	6.3 ± 0.0	1.7 ± 0.0	0.0 ± 0.0	16.3 ± 0.167	4.533 ± 0.057	22.4 ± 0.2
**FL3i**	0.0 ± 0.0	7.1 ± 0.0	29.167 ± 0.167	10.467 ± 0.057	3.233 ± 0.057	45.1 ± 0.577	20.167 ± 0.167	36.833 ± 0.1
**FL4**	0.0 ± 0.0	1.933 ± 0.057	6.3 ± 0.0	1.467 ± 0.057	0.0 ± 0.0	18.267 ± 0.167	5.367 ± 0.167	25.633 ± 0.167
**FL4i**	0 ± 0	7.8 ± 0.16	28.3 ± 0.76	10.07 ± 0.53	3.17 ± 0.09	48.6 ± 1.33	19.8 ± 0.41	37.6 ± 0.53
**FLC**	0.333 ± 0.057	2.733 ± 0.057	0.0 ± 0.0	0.7 ± 0.0	0.0 ± 0.0	18.9 ± 0.167	7.333 ± 0.057	21.833 ± 0.167
**FCLi**	0.0 ± 0.0	7.9 ± 0.0	0.0 ± 0.0	7.267 ± 0.057	0.0 ± 0.0	48.4 ± 0.833	20.567 ± 0.167	32.0 ± 0.353
